# Pleural Infiltration Revealing Post‐Essential Thrombocythemia Acute Myeloid Leukaemia

**DOI:** 10.1002/jha2.1108

**Published:** 2025-11-12

**Authors:** Clémentine Muron, Damien Luque Paz, Marie‐Christine Copin, Corentin Orvain

**Affiliations:** ^1^ Maladies du Sang CHU d'Angers Angers France; ^2^ Fédération Hospitalo‐Universitaire Grand‐Ouest Acute Leukemia FHU‐GOAL Angers France; ^3^ Université d'Angers Inserm UMR 1307 CNRS UMR 6075 Nantes Université CRCI2NA Angers France; ^4^ Laboratoire d'Hématologie CHU d'Angers Angers France; ^5^ Service de Pathologie, Université d'Angers, CHU d'Angers Angers France

1

A 73‐year‐old female patient was diagnosed with essential thrombocythemia (ET) with *CALR^mut^* and *DNMT3A^mut^* at diagnosis (Variant Allele Function [VAF] 14.3% and 31.2%, respectively). While receiving cytoreductive therapy with hydroxycarbamide for 6 years, she developed progressive respiratory failure, associated with discrete anaemia. During the diagnostic workup, she underwent positron emission tomography‐computed tomography that showed extensive pleural and peri‐hilum hypermetabolism (Figure 1, left panel). At that time, blood counts were subnormal (leukocytes, 3.46 g/L; haemoglobin, 110 g/L; platelets, 433 g/L). Bronchial fibroscopy showed a granulating infiltration at the left superior lobar bronchus entrance with biopsies showing extra‐medullary myeloid blast cells (immunohistochemistry showed CD34 [higher right image], MPO [lower right image], CD4 and CD56 expression; Figure 1, right panel; objective ×40). Bone marrow aspiration confirmed post‐ET acute myeloid leukaemia (AML) with 44% myeloid blasts and a complex karyotype including monosomy 7 and a del(5q) deletion. Next Generation Sequencing (NGS) showed persistent *CALR* and *DNMT3A* mutations, but with lower VAF (2.3% and 1.3%, respectively), while two pathogenic mutations on *NRAS* and *SETBP1* genes were observed (VAF 31% and 33%, respectively), suggesting that the leukaemic transformation evolved at the expense of a different clone than the one associated with the myeloproliferative neoplasm (MPN). Due to technical issues, we could not assess the mutational profile in the bronchial biopsy.

**FIGURE 1:**
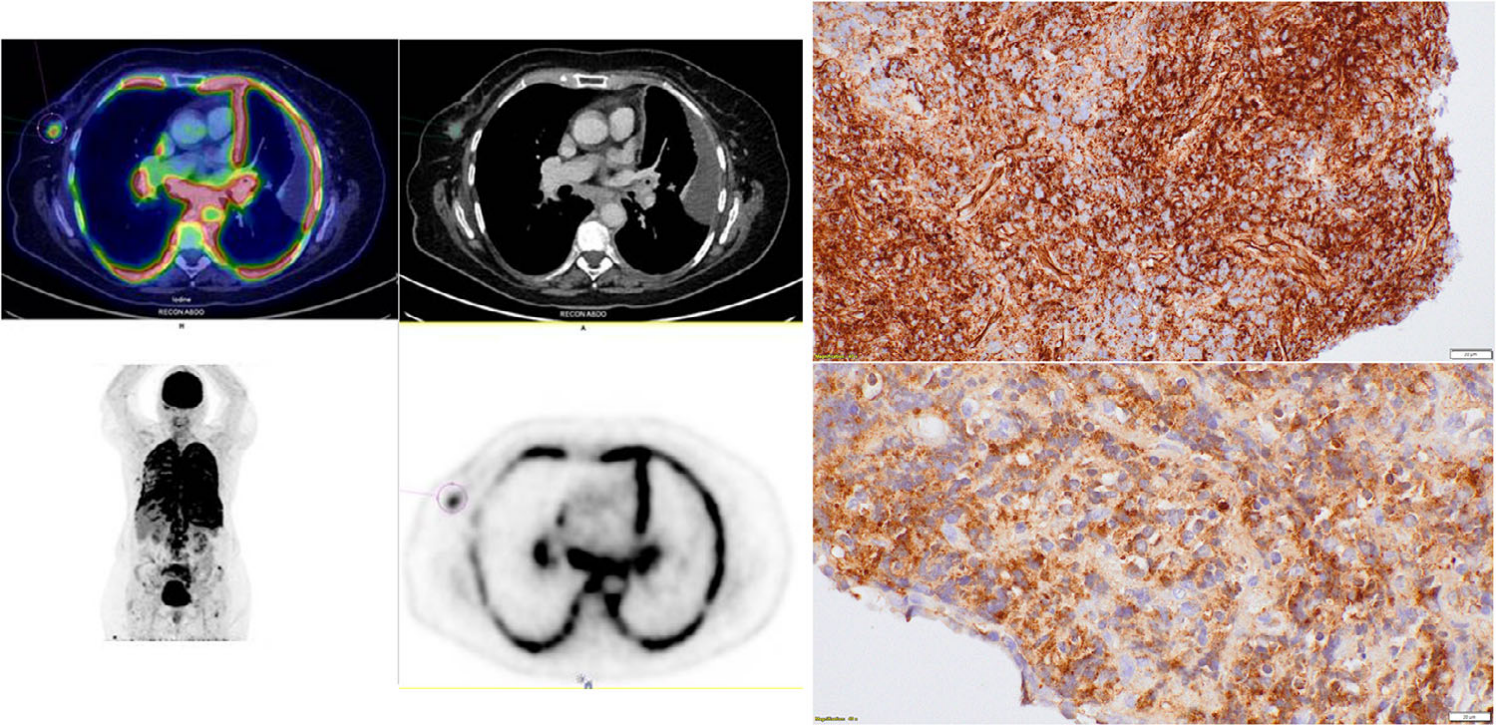
Positron emission tomography‐computed tomography (PET‐CT) imaging at diagnosis (**left panel**) showed intense hypermetabolism in pulmonary parenchyma (peri‐hilum) and pleura representative of blast cell infiltration, and bronchial histology (**right panel**) showed CD34 (upper) and MPO (lower) strong expression in blast cells infiltrating the bronchial mucosae (×40).

The patient was included in a clinical trial evaluating induction therapy with CPX‐351, a liposomal formulation of encapsulated cytarabine and daunorubicin, in patients with post‐MPN AML (NCT04992949) [1]. After one cycle, she achieved complete morphologic and cytogenetic remission but no extra‐medullary response with persistent bronchial myeloid blast cells. After one cycle of second‐line therapy with azacitidine and venetoclax [2, 3, 4], bone marrow aspiration showed very poor marrow attributable to treatment toxicity, with no excess blast; a chest scan showed partial regression of pleural effusion and clinical improvement was observed. While receiving a second cycle of azacitidine–venetoclax, she presented to the emergency room with confusion, with a brain scan and cerebrospinal fluid examination confirming tumoral meningitis. The patient declined any further therapy and eventually died from AML.

Extramedullary localization of AML (or myeloid sarcoma) is a rare presentation of post‐MPN AML that can occur without clear evidence of peripheral blood involvement by AML, as presented in this case [5]. This suggests that histopathological documentation of suspicious extramedullary lesions should be obtained in patients with MPN, even(Figure 1) with normal blood count.

## Author Contributions

Conception and design: Clémentine Muron and Corentin Orvain. Collection and assembly of data: Clémentine Muron, Damien L. Paz, Marie‐Christine Copin and Corentin Orvain. Data analysis and interpretation: Corentin Orvain, Damien L. Paz, Marie‐Christine Copin. Manuscript writing: Clémentine Muron and Corentin Orvain. Final approval of the manuscript: All authors.

## Ethics Statement

The authors have nothing to report.

## Consent

Written informed consent was obtained from the patient.

## Conflicts of Interest

The authors declare no conflicts of interest.

## Data Availability

The authors have nothing to report.
